# Integrated Temperature and Hydrogen Sensors with MEMS Technology

**DOI:** 10.3390/s18010094

**Published:** 2017-12-31

**Authors:** Hongchuan Jiang, Min Huang, Yibing Yu, Xiaoyu Tian, Xiaohui Zhao, Wanli Zhang, Jianfeng Zhang, Yifan Huang, Kun Yu

**Affiliations:** 1 State Key Laboratory of Electronic Thin Films and Integrated Devices, University of Electronic Science and Technology of China, Chengdu 610054, China; hmpine@hotmail.com (M.H.); 201621030517@std.uestc.edu.cn (Y.Y.); 201621030515@std.uestc.edu.cn (X.T.); xhzhao@uestc.edu.cn (X.Z.); wlzhang@uestc.edu.cn (W.Z.); 2 National Key Laboratory of Science and Technology on Vacuum Technology and Physics, Lanzhou Institute of Physics, Lanzhou 730000, China; zhangjianfeng510@spacechina.com (J.Z.); huangyifan@spacechina.com (Y.H.); 3 Institute of Chemical Materials, China Academy of Engineering Physics, Mianyang 621000, China; yukun@caep.cn

**Keywords:** hydrogen gas sensor, PdNi thin film, TCR, MEMS

## Abstract

In this work, a PdNi thin film hydrogen gas sensor with integrated Pt thin film temperature sensor was designed and fabricated using the micro-electro-mechanical system (MEMS) process. The integrated sensors consist of two resistors: the former, based on Pt film, is used as a temperature sensor, while the latter had the function of hydrogen sensing and is based on PdNi alloy film. The temperature coefficient of resistance (TCR) in both devices was measured and the output response of the PdNi film hydrogen sensor was calibrated based on the temperature acquired by the Pt temperature sensor. The SiN layer was deposited on top of Pt film to inhibit the hydrogen diffusion and reduce consequent disturbance on temperature measurement. The TCR of the PdNi film and the Pt film was about 0.00122/K and 0.00217/K, respectively. The performances of the PdNi film hydrogen sensor were investigated with hydrogen concentrations from 0.3% to 3% on different temperatures from 294.7 to 302.2 K. With the measured temperature of the Pt resistor and the TCR of the PdNi film, the impact of the temperature on the performances of the PdNi film hydrogen sensor was reduced. The output response, response time and recovery time of the PdNi film hydrogen sensors under the hydrogen concentration of 0.5%, 1.0%, 1.5% and 2.0% were measured at 313 K. The output response of the PdNi thin film hydrogen sensors increased with increasing hydrogen concentration while the response time and recovery time decreased. A cycling test between pure nitrogen and 3% hydrogen concentration was performed at 313 K and PdNi thin film hydrogen sensor demonstrated great repeatability in the cycling test.

## 1. Introduction

Hydrogen plays a significant role in chemical production, fuel cell technology, fuel for cars, rocket engines, nuclear reactors, etc. [1]. However, hydrogen is colorless, odorless, tasteless, flammable and explosive at concentrations above 4%. Extensive researches have been conducted to improve the output response, stability, selectivity, response time and recovery time of hydrogen sensors. Based on the working mechanism, most of the hydrogen sensors can be classified as electrochemical, electrical and optical type, et al. [2]. Among these sensors, the electrical resistor based hydrogen sensors are widely used due to advantages such as fast response, simple detection method and low power consumption, et al. [2]. 

Over the last few decades, Pd was widely used as the sensitive material for the resistor type of hydrogen sensors because of its high output response to hydrogen [1,2,3,4,5]. In spite of the high output response of Pd based hydrogen sensors, this sensor has some drawbacks. For example, pure Pd thin film is subject to microstructural deformation originated from volume expansion under high hydrogen concentration [6]. Adding other metal atoms to form palladium alloy has been proved as an effective way to improve the stability of the microstructure. R.C Hughes et al. reported that PdNi alloy thin film with Ni contents of 8 at.% and 15 at.% presented durable and quickly reversible detection of high hydrogen concentration [7]. L. Huang et al. reported that porous and discontinuous PdNi thin film could efficiently suppress blisters and respond quickly to hydrogen [8]. However, the impact of temperature on the resistance of PdNi film is not considered and the fabrication processes of the sensor are not suitable for mass production.

In this paper, a PdNi thin film hydrogen sensor integrated with a Pt thin film temperature sensor was designed and fabricated by the micro-electro-mechanical system (MEMS) technology. The Pt thin film resistor was integrated for precise measurement of the environment temperature. Additionally, the performances of PdNi thin film hydrogen sensors were characterized and discussed.

## 2. Experimental

### 2.1. Sensor Fabrication

**Figure 1:**
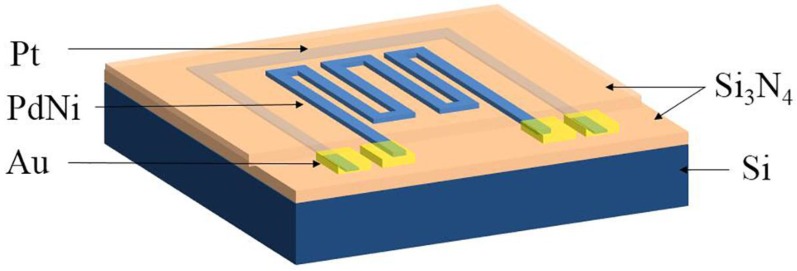
Schematic diagram of the hydrogen sensors.

The schematic diagram of the hydrogen sensor is shown in Figure 1. The sample was designed with the size of 1× 1 × 0.5 mm^3^. A silicon wafer with a silicon nitride layer was chosen as the substrate. The Pt film was deposited as the temperature resistor for precise temperature measurement, it was covered with silicon nitride layer to inhibit the hydrogen diffusion into the Pt film and reduce consequent disturbance on temperature measurement. The PdNi alloy film was deposited as the hydrogen sensing resistor. The bond pads of the PdNi and the Pt resistors were covered with a Au film for precise resistance measurement. The specific fabrication process is as follows. An n-type (100) silicon wafer (20 × 20 × 0.5 mm^3^) was used as the substrate. Before deposition, the silicon substrate was ultrasonic-cleaned sequentially by using acetone, alcohol, and deionized water for 10 min. Next, the substrate was blow dried by nitrogen gas. In order to improve the adherence of the sensor, a silicon nitride film with thickness of 50 nm was deposited on the cleaned substrate by radio frequency reactive magnetron sputtering. Then, PRI-4000A photoresist was spin-coated onto the as-deposited silicon nitride film and patterned with lithographic technology. A Pt thin film resistor was integrated close to PdNi resistor for precise temperature measurement. The Pt film with the thickness of 90 nm was deposited by DC magnetron sputtering and patterned with lift-off technique. Another silicon nitride layer with the thickness of 25 nm was deposited and patterned on the Pt resistor. Next, the Pd_85_Ni_15_ alloy film with a thickness of 100 nm was deposited by DC magnetron sputtering and patterned with a lift-off technique. A Au film with the thickness of 280 nm was deposited by DC magnetron sputtering and patterned on the bond pads of PdNi and Pt resistors. Details of all the deposition process are included in Table 1.

**Figure 2:**
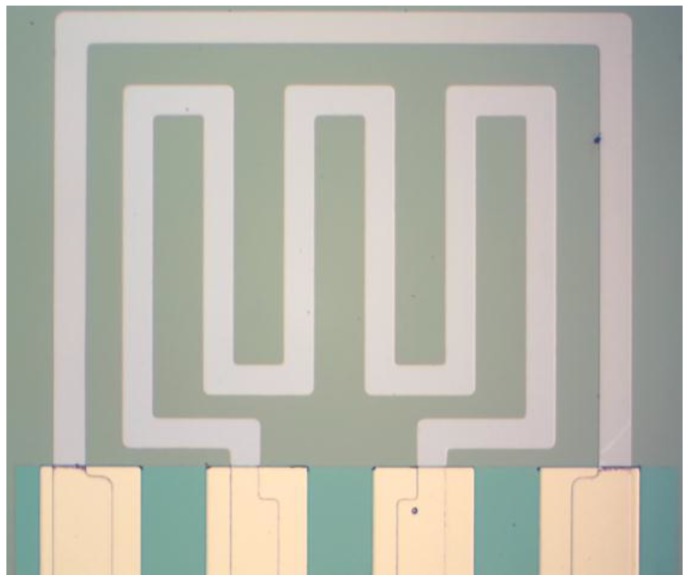
Photo of the fabricated hydrogen sensor.

After the fabrication, the sample was annealed in vacuum tube furnace at 350 ℃ for 2 h. Finally, the sample was sliced into single sensor with the size of 1 × 1 mm^2^. The photo of the fabricated hydrogen sensor is shown in Figure 2; all of the deposited films are dense and smooth with clear configuration.

### 2.2. TCR Tests

The TCR of the Pt film and the PdNi film were measured in the gas chamber, respectively. The gas chamber was wrapped by a heater belt connected with a temperature controller. Before the measurement, the chamber was purged with pure nitrogen for 4 h with a flow rate of 100 sccm. After that, the base resistance of the Pt film or PdNi film was obtained. Next, the chamber was heated from 304 K to 330 K, and the resistance of the film was measured by the Keithley 2400 Source Meter with source current of 1 mA.

### 2.3. Hydrogen Sensing Tests

**Figure 3:**
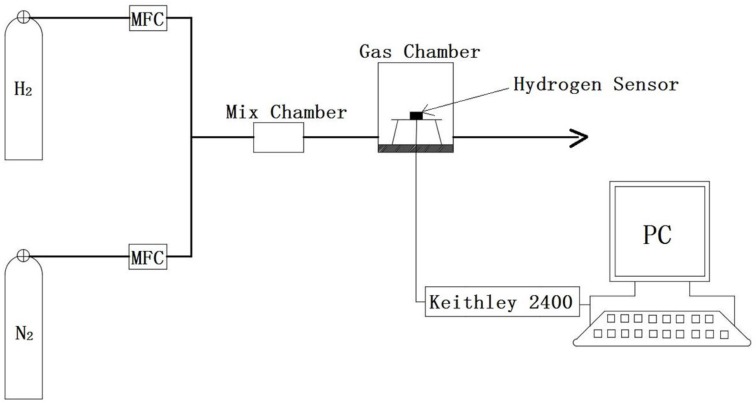
Schematic of the hydrogen measuring system.

The hydrogen measuring system is composed of mass flow controllers (MFC), gas mix chamber, gas test chamber, temperature controller and Keithley 2400 Source Meter, as shown in Figure 3. The hydrogen sensors were measured in the test chamber at atmospheric pressure, and the measurement temperature varies from 294 K to 330 K. Before the measurement, the chamber was purged with pure nitrogen for 4 h with a flow rate of 100 sccm and the base resistance of the PdNi film was obtained. Next, the gas mixture of nitrogen and hydrogen with different amount of hydrogen was introduced into the gas mix chamber. After mixing, the gas mixture was delivered to the test chamber with a constant flow rate of 100 sccm. The resistances of the PdNi thin film hydrogen sensor and the Pt thin film resistor were acquired with a LabVIEW program (National Instruments) through a Keithley 2400 Source Meter under constant current with source current of 1 mA. The output response (*R_S_*) is defined as formula (1) [9]:

(1) $$R_{S}\left(\%\right)=\frac{R_{H}-R_{0}}{R_{0}}\times 100=\frac{\Delta R}{R_{0}}\times 100$$

where *R*_0_ and *R_H_* were the base resistance and the resistance in the gas of interest, respectively.

## 3. Results and Discussions

**Figure 4:**
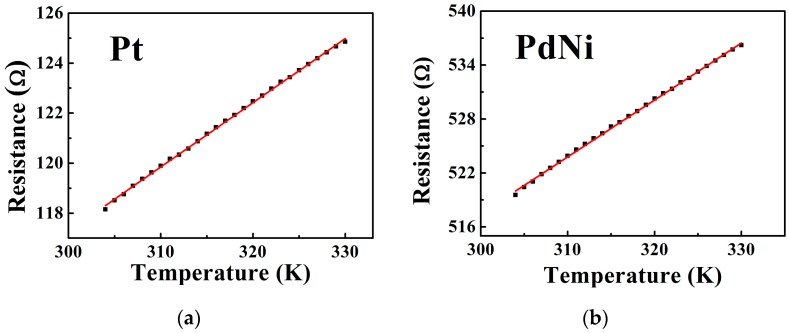
The resistance variations of the Pt (**a**) film and PdNi (**b**) film with temperatures.

Figure 4 shows the variations of the Pt film and the PdNi film resistance with the temperature. The TCR of the PdNi film and the Pt film calculated from Figure 4 were 0.00122/K and 0.00217/K, and the resistance at 304 K were used as *R* in the calculation of TCR. Apparently, the TCR of the PdNi film is not negligible, so the temperature correction of the output of the sensor is necessary to reduce the influence of the temperature fluctuation during practical application.

**Figure 5:**
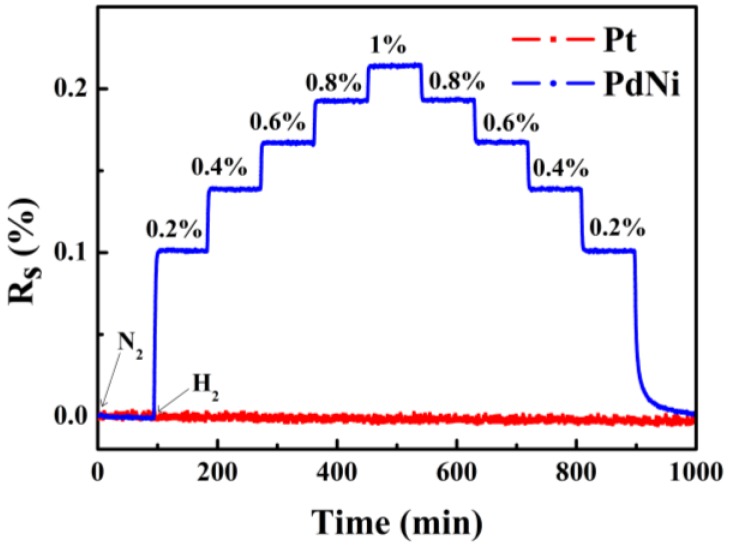
Response curves of the Pt film and PdNi film under various H_2_ concentrations.

The output response of the Pt film (shielded with SiN) and the PdNi film under various hydrogen concentration were measured at 318 K, as shown in Figure 5. Compared to the obvious variation of the output response of the PdNi film with a different hydrogen concentration, the resistance of Pt film is quite consistent, suggesting that as prepared SiN layer is thick enough to shield the Pt film from hydrogen. By this way, the precise measurement of temperature is guaranteed.

**Figure 6:**
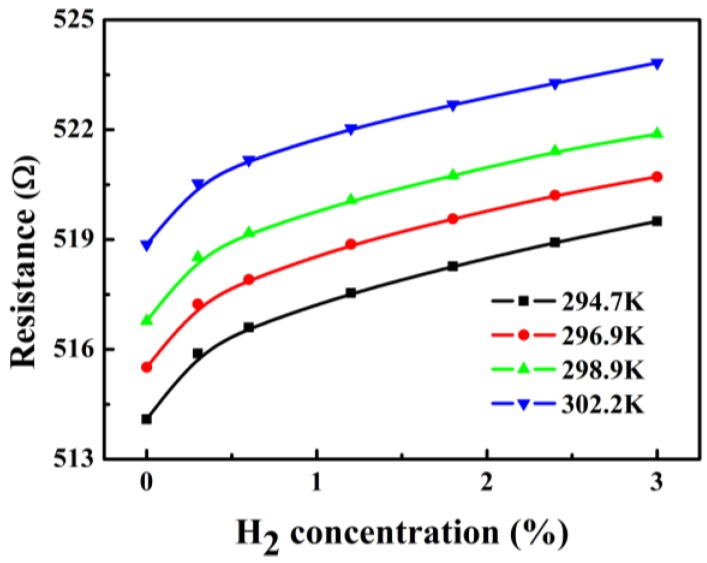
The variations of the resistance of the PdNi film with H_2_ concentrations measured at different temperatures.

The resistance of the PdNi film with hydrogen concentrations were measured at different temperatures, as shown in Figure 6. Under certain measured temperature, the resistance of the PdNi film tended to increase with the increasing hydrogen concentration. This could be ascribed to the adsorption of hydrogen molecules on the PdNi film. When a hydrogen molecule is absorbed on the surface of the PdNi film, the hydrogen molecule decomposes into hydrogen atoms and then diffuse into the PdNi thin film. These absorbed hydrogen atoms tend to react with Pd atoms to form palladium hydride (PdH_x_) and lead to resistance increment of the PdNi film. When the hydrogen concentration is relatively low, there are plenty of Pd atoms on the surface of the alloy film to react with, more hydride could form with increasing hydrogen concentration, leading to linear increase of the resistance of the PdNi film [10,11]. Moreover, as precious metal, Pd is inert to the moisture, O_2_ and other interfering gas at ambient temperature, resulting in excellent selectivity of the sensor [12].

**Figure 7:**
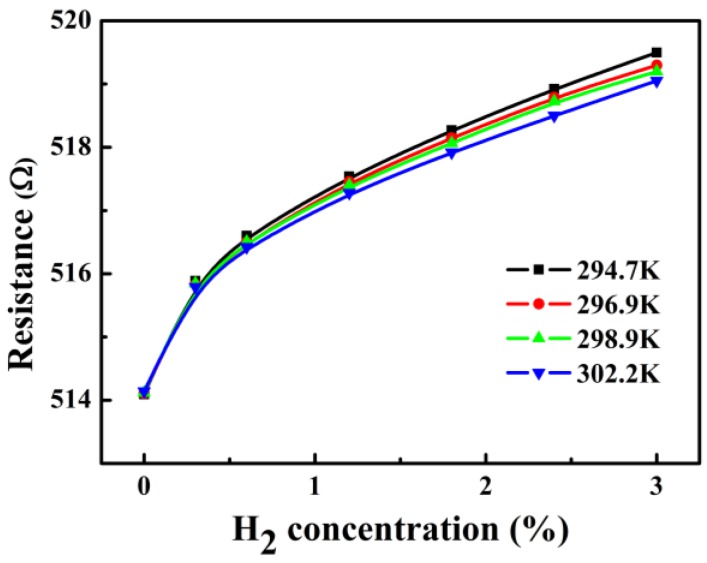
Resistances of the PdNi film under various H_2_ concentrations with the temperature correction.

Due to the TCR of the PdNi film, the base resistance of PdNi film shifts to higher values with increasing measurement temperatures. Based on the TCR results above, a temperature correction of the output response of the hydrogen sensor is performed. The temperature of 294.7 K was defined as the standard operating temperature of the sensor, while the resistance measured at other temperature could be modified according to formula (2):

(2) $$R^{*}=\frac{R}{1+\left(T-T_{0}\right)\times \mathrm{TCR}}$$

where *T*_0_ is the standard operating temperature, *R* is the measured resistance, *T* is the temperature measured from the Pt resistor, and R* is the corrected resistance. The variations of the resistance of the PdNi film after the temperature correction are shown in Figure 7. The resistances are quite consistent at low hydrogen concentration range and the fluctuation of the resistance is no more than 0.1%, even when the hydrogen concentration reaches 3%. This result indicates that after the temperature correction, the fabricated sensor demonstrates excellent reliability in real time hydrogen measurements despite of temperature fluctuations. There are still some deviations at high hydrogen concentration range, and this could be well illustrated by Sieverts’ law, which claims that the logarithm of hydrogen solubility increases linearly with inverse temperature under a given hydrogen partial pressure [1,3,13].

**Figure 8:**
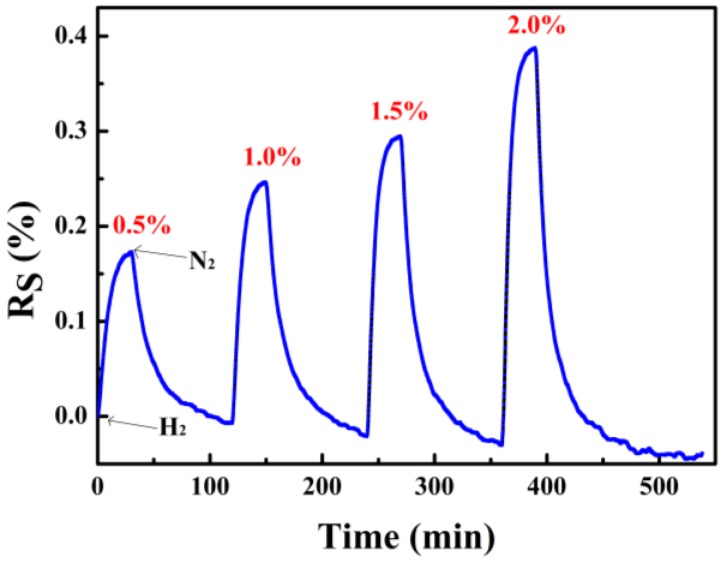
Response curve of the PdNi film hydrogen sensor under different H_2_ concentrations.

Figure 8 shows the variations of resistance of the sensor under pure nitrogen and 0.5, 1.0, 1.5 and 2.0% hydrogen concentrations at 313 K. The resistance of the PdNi film increased rapidly after the inlet of the hydrogen and decreased after purging with nitrogen. Compared with the sensor on alumina substrate, the output response is relatively lower than the reported results [8,10]. We believe this is certainly related with the configuration of the hydrogen sensor. In this work, in order to improve the reproducibility of the sensor, silicon wafer was used as the substrate. Compared with those sensors fabricated on porous alumina substrates, the specific surface area of the deposited PdNi film decreased, resulting in less chance of hydrogen absorption and low permeability [8,13].

**Figure 9:**
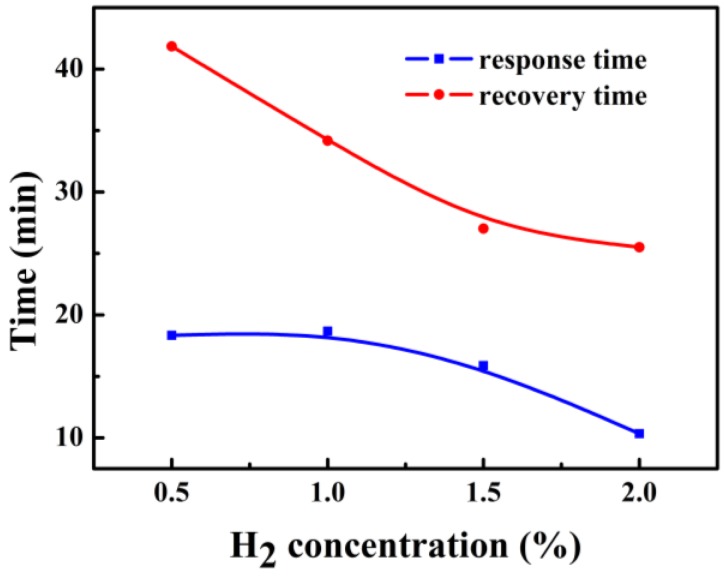
The response time and the recovery time of the PdNi film sensor.

Figure 9 shows the response time and recovery time of the PdNi film hydrogen sensor under different hydrogen concentrations. The response time and recovery time are defined as the elapsed time to reach 90% of the final equilibrium value [14]. Both the response time and recovery time are decreased with increasing hydrogen concentrations. It demonstrates that the reaction rate of both the forward reaction and the reverse reaction of the hydride formation increased with increasing hydrogen concentrations. The response time is in minutes, and we believe this is related with the large volume of the gas chamber. A longer time is needed to finish the gas replacement, leading to an increased response time of the sensors.

**Figure 10:**
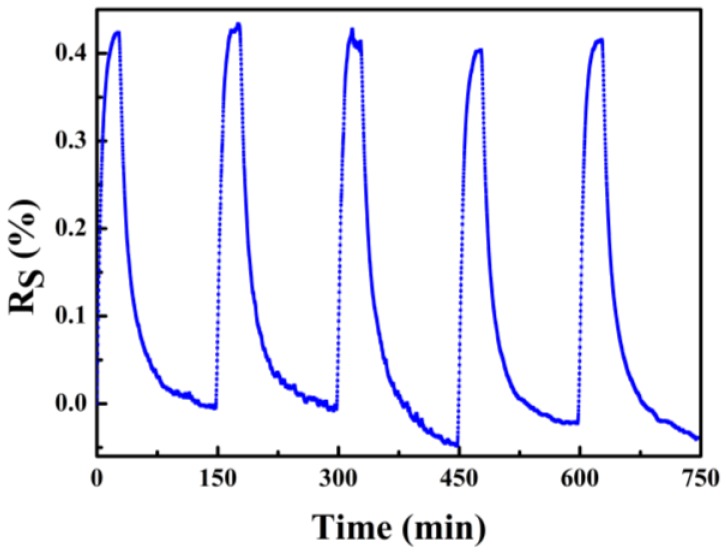
Five cycles of the PdNi film resistor under 3% H_2_ concentration.

A typical cycling response for the hydrogen sensor under 3% hydrogen concentration is shown in Figure 10. When the hydrogen was introduced into the test chamber, the output response increased with the time. After the hydrogen was shut down, the output response decreased sharply. This PdNi thin film hydrogen sensor shows good repeatability and the fluctuations of the output response under 3% hydrogen concentration are less than 3.2%. This can be ascribed to the composition and microstructure of the PdNi composite film. After the addition of Ni in the Pd film, the irreversible surface deformation of the pure Pd film is suppressed [10]. Although the solubility of hydrogen is reduced, the stability of the hydrogen absorption is enhanced, leading to the improvement of the repeatability of the PdNi film hydrogen sensor. The baseline drift can be observed in Figure 8 and Figure 10, which has been reported previously and can be ascribed to the grain size variation of PdNi film [15].

Compared with the state-of-the art Pd and PdAg hydrogen sensor, although the output response is relatively smaller, the repeatability of the sensor is similar [13,16]. Particularly, the size of this MEMS based hydrogen sensor is much smaller and easy for integration. To our knowledge, this is the first time that a temperature sensor was integrated and the related temperature correction was performed [1,8,10].

## 4. Conclusions

In this paper, a PdNi thin film hydrogen sensor was designed and fabricated by a magnetron sputtering and MEMS process. A Pt thin film resistor was integrated as the temperature sensor to reduce the influence of temperature on the performance of the PdNi thin film hydrogen sensor. With the shield of SiN layer, precise temperature measurement with Pt thin film resistor is achieved. The temperature correction was performed and the influence of temperature fluctuation on the hydrogen sensor sufficiently reduced. Moreover, the irreversible surface deformation of the pure Pd film was suppressed by the addition of Ni. As a result, the output response of the PdNi thin film hydrogen sensor demonstrates excellent repeatability during cycling tests. This integrated sensor is small, compact and can be applied in circumstances with temperature variations.

## Figures and Tables

**Table 1:** Sputtering parameters of different functional film.

Material	Base Pressure (Pa)	Sputtering Pressure (Pa)	Sputtering Power (W)	Temperature (°C)
Pt	8 × 10^−4^	0.4	80	RT
Si_3_N_4_	8 × 10^−4^	0.5	200	RT
PdNi	8 × 10^−4^	0.3	60	RT
Au	8 × 10^−4^	0.3	60	RT
